# Impact of storage conditions on the fracture reliability and physical properties of a dental resin-based composite

**DOI:** 10.1590/1807-3107bor-2024.vol38.0062

**Published:** 2024-07-15

**Authors:** Afnan Omar AL-ZAIN, Evandro PIVA, Alice Hammes PIMENTEL, Camila Gonçalves DUARTE, Lisia Lorea VALENTE, Cristina Pereira ISOLAN, Eliseu Aldrighi MÜNCHOW

**Affiliations:** (a) King Abdulaziz University, Faculty of Dentistry, Restorative Dentistry Department, Jeddah, Saudi Arabia; (b) Universidade Federal de Pelotas – UFPel, Developmental and Control Center of Biomaterials, Department of Restorative Dentistry, Pelotas, RS. Brazil; (c) Universidade Federal do Rio Grande do Sul – UFRGS, School of Dentistry, Graduate Program in Dentistry, Porto Alegre, RS, Brazil.; (d) Universidade Federal dos Vales do Jequitinhonha e Mucuri – UFCJM, School of Biological and Health Sciences, Department of Dentistry, Diamantina, MG. Brazil.; (e) Universidade Federal do Rio Grande do Sul – UFRGS, School of Dentistry, Department of Conservative Dentistry, Porto Alegre, RS, Brazil.

**Keywords:** Composite Resins, Hardness Tests, Flexural Strenght, Spectrum Analisys

## Abstract

This study investigated the impact of ‘storage condition’ and ‘period of storage’ on selected physico-mechanical properties and fracture reliability of a resin-based composite (RBC). Specimens, prepared from a nanofilled RBC (Filtek Z350 XT; 3M ESPE), underwent tests for degree of conversion (DC), flexural strength (σ), flexural modulus (E), and hardness. The specimens were initially grouped into dry storage at 37°C or wet storage in distilled water at 37°C. Subsequently, they were further divided into four subgroups based on the period of storage: 6, 24, 72, or 168 hours. Specimens tested immediately after preparation served as control. Data analysis employed two-way ANOVA and Weibull analysis (α = 5%). Compared to the control, an increase in DC was observed only after 72 hours of dry storage; σ showed higher values after both dry and wet storage, regardless of the storage period (except for the group wet-stored for 168 hours); E increased with dry storage for at least 24 hours or wet storage for 72 hours; and hardness increased after dry storage for at least 24 hours or wet storage for up to 72 hours. The Weibull modulus remained unchanged under any of the distinct storage conditions. Dry storage resulted in greater characteristic strength than the control, whereas wet storage contributed to higher strength values only at shorter periods (up to 24 hours). Overall, the inherent properties of RBCs with a similar composition to that tested in this study may change with varying storage conditions and periods.

## Introduction

Laboratory tests are commonly conducted to evaluate the in vitro performance of dental biomaterials.^1-3^ While in vitro tests may not entirely replicate real oral conditions, they are considered reliable and important sources for measuring and predicting material behavior under clinical use.^4^ It is well-established that these laboratory tests should adhere to standardized conditions, and dentistry relies on various international standards (e.g., ISO 4049, NIST No. 4877) to guide researchers, facilitating comparisons across studies.^1,2,5^ According to Bona et al.’s 2012 study,^5^ which examined the use of standards in articles published in international dental journals over a 5-year period, ISO 4049 was among the most frequently reported standards.^6^ This particular standard addresses dental polymer-based filling, restorative, and luting materials, providing technical requirements for the preparation of resin-based specimens in various tests and methodologies.^6^

Despite serving as a guide for specimen preparation and test conduction, ISO 4049 and other standards specify a chronological schedule for specimen preparation.^6^ Interestingly, one parameter for preparing resin-based composite (RBC) specimens involves storing them in distilled water at 37°C until testing. Notably, a 24-hour storage period is typically reported by researchers worldwide, although it is not explicitly detailed in ISO 4049.^6^ Moreover, there is a notable gap in the literature regarding the potential compromise of laboratory tests if the specified storage condition protocol (i.e., wet storage for 24 hours) is not strictly followed. Is it then conceivable that altering the storage time—making it either shorter or longer—could significantly modify the material properties? To the best of our knowledge, there is still insufficient information on this aspect, warranting further investigation.

Therefore, this in vitro study aimed to assess the impact of storage conditions (specifically, ‘period of storage’ and ‘storage condition’) on selected physico-mechanical properties of a dental light-cured RBC. Additionally, we applied Weibull statistics to gain deeper insights into the effects of various storage conditions on the material’s reliability.^7, 8^ The null hypothesis posited that the different storage conditions would not influence the overall properties nor the reliability of the RBC.

## Methods

### Experimental design

This in vitro 2 × 4 factorial study (n = 20) assessed two variable factors: storage condition (dry storage at 37°C vs. wet storage in distilled water at 37°C) and the period of storage until testing (immediate testing or after 6, 24, 72, or 168 hours). We utilized a nanofilled RBC (Filtek Z350 XT; 3M ESPE, St. Paul, USA). The response variables included degree of conversion (DC), flexural strength (σ), flexural modulus (E), hardness (KMN), Weibull modulus (m), and characteristic strength (σ_0_). Specimens tested immediately after preparation were designated as the reference group (control).

### Physico-mechanical characterization

For DC analysis, a metallic mold (6 mm diameter × 1 mm thickness) was centrally positioned over the Attenuated Total Reflection (ATR) diamond crystal of an infrared spectrometer (Prestige21, Shimadzu, Japan). The RBC was placed inside the mold and pressed flat against glass to achieve uniform thickness. A baseline analysis was obtained before photo-activation by ATR Fourier Transform Infrared (FTIR) in absorbance mode under the following parameters: 24 scans, range within 1750 and 1550 cm^-1^, resolution of 8 cm^-1^, at 25°C ± 1°C. Subsequently, each specimen was photo-activated with a single-emission peak light-emitting diode (LED) curing unit (Radii^®^; SDI, Australia – irradiance of 900 mW/cm^2^) for 20 seconds at a standardized distance of 0 mm from the specimen top.

In total, 40 specimens were prepared and allocated into two groups based on the storage condition (n = 20): dry storage in an empty opaque vial or wet storage in distilled water, both conditions at 37°C. ATR spectra were acquired immediately after photo-activation and after 6, 24, 72, and 168 hours since photo-activation. After each time point, the specimens were measured again using FTIR. The DC was calculated as described elsewhere^3^ based on the intensity of the carbon-carbon double-bond stretching vibrations (peak height) at 1635 cm^-1,^ using symmetric ring stretching at 1610 cm^-1^ from the polymerized and non-polymerized samples as an internal standard. The formula (Eq. 1) used to calculate the DC was as follows:

Eq. 1:


$$DC=1-\frac{\left(1635{cm}^{-1}÷1608{cm}^{-1}\right)\text{ cured }}{\left(1635{cm}^{-1}÷1608{cm}^{-1}\right)\text{ uncured }}\times 100\%$$

For the flexural strength and flexural modulus properties, the ISO 4049:2019 served as a guide for specimen fabrication and test conduction. However, there was a difference in terms of specimen size, with a smaller dimension used, compared to what is commonly practiced in prior studies.^9, 10^ Ninety bar-shaped specimens (12 mm length × 2 mm width × 2 mm thickness) were prepared using a metallic mold. Photo-activation was performed with the LED unit for 20 seconds on both the top and bottom surfaces.

Ten specimens were tested immediately after photo-activation using a three-point bend test in a universal testing machine (DL500; Emic, São José dos Pinhais, Brazil). The test utilized a support distance of 8 mm and a crosshead speed of 1 mm/min.^9^ The remaining specimens (n = 10) were allocated to the various storage conditions previously described and subjected to the three-point bend test after their respective storage periods.

Flexural strength (σ) and flexural modulus (E) were calculated using the following formulas (Eq. 2 and Eq. 3), expressed in MPa and GPa, respectively:


$$\text{ Eq. 2: }\sigma =\frac{3Fl}{3bh^{2}}$$

$$\text{ Eq. 3: }E=\frac{Fl^{3}}{4bh^{3}d}$$

Here, F is the peak load (N); l is the span length (mm); b is the width of the specimen (mm), h is the thickness of the specimen (mm), and d is the deflection of the specimen at load F1 during the straightest line portion of the load-displacement trace.

Following the three-point bend test, the fractured specimens were embedded in epoxy resin and wet-finished with #600- and #1200-grit silicon carbide papers. Measurements were conducted using a Knoop microhardness tester (FM 700; Future Tech, Japan) with a 50 g load and a 15-second dwell time.^3^ The Knoop Microhardness Number (KNM) was then calculated as the ratio of the load applied by the indenter to the unrecovered projected area. Five indentations were performed on each specimen and the values were averaged.

### Statistical analysis

Data were analyzed using SigmaPlot 12.0 (Systat Software Inc., San Jose, USA). For DC data, two-way Repeated Measures Analysis of Variance (ANOVA) and Tukey tests were employed to assess the effects of different storage conditions. The data for σ, E, and KMN underwent two-way ANOVA and Tukey analysis. Each experimental group was also compared to the reference group (immediate testing) using t-tests or paired t-tests. Weibull analysis was conducted to determine the fracture reliability of specimens tested in the three-point bend test, investigating the^11^ Weibull modulus (m) and the characteristic strength (σ_0_) parameters. The significance level was set at α = 5%.

## Results

Table presents the results for the DC, σ, E, KMN, m, and σ_0_ properties tested in the study. In comparison to the reference group, DC increased only after 72 hours of dry storage; σ was higher after both dry and wet storage, regardless of the storage period (except for the group wet-stored for 168 hours); E increased with dry storage for at least 24 hours or wet storage for 72 hours; and hardness increased after dry storage for at least 24 hours or wet storage for up to 72 hours. The Weibull modulus remained unchanged under all distinct storage conditions in the study. Dry storage led to greater σ_0_ than the reference group, regardless of the storage period, whereas wet storage contributed to higher σ_0_ values only at shorter periods (up to 24 hours).

**Table:** Results for the properties investigated in the study: degree of conversion (DC), flexural strength (σ), flexural modulus (*E*), Knoop microhardness number (KMN), Weibull modulus (*m*), and characteristic strength (σ0).

Properties tested	Storage condition	Period of storage (hours)				Reference group

		6	24	72	168	
DC, %	Dry	62.9 (5.8) ^A, b^	64.1 (4.8) ^A, b^	**73.1 (2.5)** ^A, a^	64.7 (5.3) ^A, b^	61.1 (4.6)
	Wet	63.6 (3.9) ^A, ab^	63.9 (3.6) ^A, a^	58.9 (5.1) ^B, bc^	56.2 (6.0) ^B, c^	
σ, MPa	Dry	**128.4 (12.2)** ^A, b^	**141.4 (14.1)** ^A, ab^	**124.5 (13.9)** ^A, b^	**152.6 (26.3)** ^A, a^	101.5 (12.6)
	Wet	**133.5 (10.6)** ^A, a^	**130.0 (14.8)** ^A, a^	**122.3 (23.1)** ^A, a^	100.1 (18.9) ^B, b^	
*E*, GPa	Dry	3.4 (0.4) ^A, c^	**4.1 (0.4)** ^A, b^	**3.8 (0.4)** ^A, bc^	**4.9 (0.8)** ^A, a^	3.1 (0.5)
	Wet	3.2 (0.7) ^A, b^	3.6 (0.5) ^B, ab^	**4.1 (0.5)** ^A, a^	3.5 (0.4) ^B, b^	
KMN	Dry	84.6 (6.3) ^B, c^	**102.6 (11.8)** ^A, b^	**102.7 (5.4)** ^A, b^	**130.4 (14.2)** ^A, a^	83.1 (9.3)
	Wet	**125.9 (13.0)** ^A, a^	**116.4 (13.5)** ^A, b^	**94.8 (4.6)** ^B, bc^	86.8 (5.9) ^B, c^	
*m*	Dry	10.1 (7.3–17.4) ^A, a^	9.6 (7.0–16.5) ^A, a^	9.0 (6.6–15.6) ^A, a^	5.3 (3.9–9.2) ^A, a^	8.1 (5.9–14.0)
	Wet	12.6 (9.2–21.7) ^A, a^	8.9 (6.5–15.3) ^A, a^	4.2 (3.0–7.2) ^A, a^	4.9 (3.6–8.5) ^A, a^	
σ_0_, MPa	Dry	**127.1 (122–137)** ^A, c^	**144.4 (139–157)** ^A, b^	**129.2 (124–141)** ^A, bc^	**175.9 (164–204)** ^A, a^	105.1 (101–116)
	Wet	**140.2 (137–150)** ^A, a^	**134.0 (128–146)** ^A, a^	**126.1 (117–152)** ^A, b^	108.3 (100–127) ^B, b^	

Different uppercase letters within the same column indicate statistically significant differences between the storage conditions, whereas different lowercase letters within the same row indicate statistically significant differences among the varying storage periods (Two-Way Repeated Measures ANOVA for DC data and Two-Way ANOVA for σ, *E*, and KMN data; p < 0.05).Mean (standard deviation) and/or median (minimum – maximum) values in bold indicate that the respective group was statistically different from the reference group (paired t-tests for DC data and t-tests for σ, *E*, and KMN data; p < 0.05).

The variations in DC, σ, E, and KMN properties relative to the period of storage under both dry and wet conditions are shown in Figure. Concerning storage conditions, dry storage yielded higher DC than wet storage after 72 and 168 hours (p < 0.001); increased σ after 168 hours (p < 0.001); elevated E after 24 hours (p = 0.026) and 168 hours (p < 0.001); reduced hardness at the 6-hour mark (p < 0.001) and increased hardness after 72 hours (p = 0.014) and 168 hours (p < 0.001). Dry storage resulted in a similar Weibull modulus compared to wet storage (p > 0.05), but a higher σ_0_ after 168 hours (p < 0.05).

**Figure:**
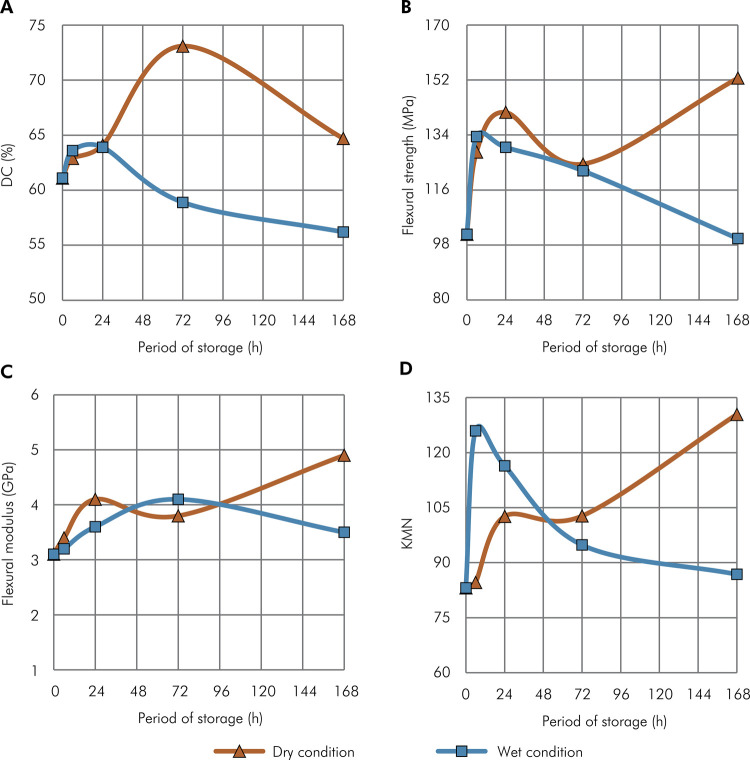
Graphs showing the alterations of the physico-mechanical properties of the resin composite under dry and wet storage conditions as a function of the storage period. (a) Degree of conversion (DC); (b) flexural strength; (c) flexural modulus; and (d) Knoop microhardness number (KMN).

Considering the effects of the storage period, DC was higher at 72 hours in dry conditions (p < 0.001) and lower after wet storage exceeding 72 hours (p ≤ 0.005). Strength properties generally increased with time during dry storage, except for specimens stored in wet conditions for 168 hours, which exhibited reduced E values. Specimens stored in dry conditions became harder over time, while those immersed in wet conditions became progressively softer with increased storage time. Weibull modulus did not vary with extended storage periods for both dry and wet conditions. However, specimens stored dry became stronger (higher σ_0_ values) over time, while those stored wet became less strong with longer storage periods. Wet storage for 168 hours resulted in the weakest behavior in terms of fracture reliability.

## Discussion

Dental RBCs are polymer-based materials that undergo significant improvements in properties upon polymerization. However, post-cure, additional conversion of unsaturated C═C double bonds into more stable and saturated C─C bonds may occur, altering inherent material properties such as strength and fracture susceptibility.^12^ Recognizing this, the need for waiting a certain period before material testing becomes justifiable, even though there is limited knowledge in the literature on this topic. It remains unclear whether waiting before testing specimens might lead to inaccurate measurements of the material’s true properties or if testing samples after a certain amount of time significantly alters the material compared to its immediate state. Hence, our study aimed to determine whether different storage conditions, such as the storage medium (dry vs. wet) and the total storage period (immediate testing vs. varying periods after polymerization) would influence selected physico-mechanical properties of a nanofilled dental RBC.

High DC values were observed only after 72 hours of dry storage. Nevertheless, RBC specimens typically undergo in vitro testing in wet conditions in distilled water for 24 hours. According to our findings, DC remained similar compared to the immediate reference group, confirming that waiting this amount of time before testing does not compromise results. It is worth mentioning that the increased DC noted after 72 hours of dry storage is likely a consequence of weak molecular interactions forming between residual unsaturated C═C double bonds in close proximity during post-cure vitrification of the material.^13^ This may explain the lower measurement of C═C double bonds during DC analysis. However, with the passage of time, this apparent gain in monomer conversion was no longer identified, possibly due to the breakage of weak bonds. In wet-stored specimens, DC tended to decrease over time, potentially owing to hydrolysis, softening the intermolecular bonds of the polymer system,^14,15^ thereby reducing the polymerization level of the material. As a result, it is then advisable to avoid wet storage for specimens longer than 24 hours when assessing the inherent material properties.

The current findings reveal that dry storage for more than 72 hours enhances the mechanical strength and hardness of the RBC, particularly after 7 days (168 hours) of storage. It is plausible to suggest that the material’s kinetic activity increases promptly after the initiation of polymerization, leading to the accumulation of energy within its bulk. This phenomenon occurs as the organic phase of the RBC undergoes rearrangement to form the polymeric network.^16,17^ Consequently, the intrinsic volumetric shrinkage of the material can generate internal stress,^18^ potentially affecting the immediate cohesiveness of the material. Considering that strength is an inherent property of RBCs that depends on compositional factors, such as the inorganic filler phase,^19-21^ the stress relief and dissipation of any accumulated energy after the post-cure may facilitate strengthening and establish a more suitable linkage between fillers and the organic phase.^13^ This is evidenced by the significant enhancement in strength properties and hardness, as indicated in Table. Therefore, it is suggested that researchers wait for a minimum period of at least 6 hours before testing the specimens.

In summary, dry storage appears to be beneficial for the properties investigated in our study, likely due to the absence of water uptake within the material’s bulk.^15^ We designed the ‘dry group’ to carefully observe differences arising from varying environmental conditions that could impact the material’s performance. However, a dry condition may not fully replicate the clinical scenario in which dental RBCs are placed. The ‘wet group’, on the other hand, better simulates in vitro material testing. Nonetheless, prolonged wet storage negatively influenced the tested properties, emphasizing that the widely adopted period of 24 hours appears to be more appropriate for storing specimens before testing, aligning with the practices of most studies conducted worldwide.

The primary objective of dental restoratives is to endure stress and to resist fracture during oral function. Our study assessed the fracture susceptibility of the tested specimens through Weibull analysis, a crucial measure for evaluating the overall reliability of a material. The strength of brittle materials (e.g., the RBC tested here) is commonly controlled by randomly distributed defects within the specimen’s volume.^19^ Our results demonstrated that, in terms of the Weibull modulus, all groups performed similarly, likely due to the use of the same restorative material, so the defects that can cause fracture were evenly distributed over the entire volume of all specimens.

In the case of light-cured RBCs, the occurrence of defects is mainly associated with the material’s handling characteristics—its ease of application into a mold or tooth cavity.^11^ For instance, sticky materials tend to result in more defective restorations filled with voids compared to less viscous materials.^22^ Since the groups in this study varied in storage period and condition but not in the type or the viscosity of the restorative material, it is plausible that the Weibull modulus parameters did not significantly differ among the groups.

However, the ‘characteristic strength’ parameter varied in groups stored for some duration compared to the non-stored reference group. Characteristic strength is a measure of the material’s reliability concerning its structure, where higher strength indicates better cohesiveness and bulk structure. In addition, the characteristic strength may largely depend on the surface finish state of the RBC.^23^ Dry or wet storage for at least 6 hours increased the characteristic strength of our specimens, indicating that some time is needed for the restoration to establish a stable polymeric network. This stable structure persisted for as long as 7 days in dry storage; conversely, wet storage for 7 days reverted the strength values of the RBC to a pattern similar to the reference group. Therefore, we infer that prolonged wet storage alters the fracture reliability of an RBC with a composition similar to the one tested here. It is advisable to test specimens earlier, but after waiting a minimum period, regardless of the storage condition.

While our study focused on comparing the effects of storage conditions and periods on the performance of a nanofilled RBC, our findings have implications in a clinical setting. The mechanical behavior of RBC restorations in clinical scenarios can exhibit changes even after brief periods of wet storage,^24^ emphasizing the importance of selecting appropriate materials to ensure durable tooth restorations. It is equally crucial for researchers to standardize their studies, particularly in terms of the medium used to simulate oral aging and the periods employed during in vitro biomaterial testing. These variables can significantly impact the overall mechanisms involved in the degradation of structural components and the stability of materials during oral function. Future studies should delve into a comprehensive fractographic analysis of various classes of RBCs tested under the same variables explored in this study.^19^

The present study considered a specimen geometry for the flexural strength test deviated from the recommendations in ISO 4049:2019.^6^ Notably, we prepared specimens with a much shorter length (*i.e.*, 12 mm), conducting the mechanical test with a span distance of 8 mm only.^9^ According to the study by Ilie,^1^ the use of shorter specimens in flexural strength testing reduces the effective volume under load, potentially yielding enhanced strength values compared to other geometries (e.g., longer specimens tested under higher spans). Despite this, the reduction in the effective volume under load to dimensions akin to clinical applications in tooth cavities is considered more relevant. This approach aligns with the use of Weibull statistics, aiding in the calculation of the reliability of the final restoration.^1^

One important limitation of our study is the use of only ten specimens per group in the flexural strength analysis, whose data were applied in the Weibull distribution. Acknowledging that n = 10 is at the lower limit for a Weibull analysis,^8^ it is imperative to interpret our results cautiously. However, the coefficient of variation of the flexural strength data ranged from 9% to 19% in the experimental groups (Table), resulting in a normal distribution with equally distributed data. In light of this, we may assume that our Weibull distribution results align with those from a normal distribution, as suggested by the study conducted by Yang et al..^25^ Despite the limited number of data points, valuable information can still be derived from the present Weibull analysis.

Additionally, another limitation of our study was the use of only one type of RBC, particularly one with a nanofilled distribution of fillers—a less common classification in the dental market. However, the same material tested here is widely employed in in vitro studies examining the performance of dental RBCs. Therefore, the effects of the different storage conditions investigated in the present study should not be extrapolated to other types of restorative materials. Minimal differences in organic matrix and filler composition can significantly alter the material’s response to mechanical loading and surface evaluation.^24^ Consequently, further studies testing different classifications of resin-based composites should be conducted using a methodology similar to ours.

Finally, our findings led to the rejection of the null hypothesis, as the tested storage conditions and periods had a significant influence on some physico-mechanical properties and the characteristic strength parameter of the nanofilled resin-based composite.

## Conclusions

In light of the potential alterations in the inherent properties of dental resin composites under different storage periods and conditions, it is recommended to wait for a minimum period of 6 hours before conducting in vitro testing. Moreover, it is advisable not to store these composites in wet conditions for longer than 24 hours. Doing otherwise may result in substantial changes in properties, impeding a proper evaluation of the unaged state of resin-based composites.
